# Effect of Probiotics on the Glucose Levels of Pregnant Women: A Meta-Analysis of Randomized Controlled Trials

**DOI:** 10.3390/medicina54050077

**Published:** 2018-11-01

**Authors:** Tzu-Rong Peng, Ta-Wei Wu, You-Chen Chao

**Affiliations:** 1 Department of Pharmacy, Taipei Tzu Chi Hospital, Buddhist Tzu Chi Medical Foundation, New Taipei City 231, Taiwan; tzu.rong@tzuchi.com.tw (T.-R.P.); p8561825@yahoo.com.tw (T.-W.W.); 2 School of Pharmacy, College of Pharmacy, Taipei Medical University, Taipei 110, Taiwan; 3 Division of Gastroenterology, Department of Internal Medicine, Taipei Tzu Chi Hospital, Buddhist Tzu Chi Medical Foundation, New Taipei City 231, Taiwan; 4 School of Medicine, Tzu Chi University, Hualien 970, Taiwan

**Keywords:** probiotics, pregnant women, fasting blood glucose, gestational diabetes, insulin concentration

## Abstract

*Background:* Gestational diabetes mellitus (GDM) is a condition, in which women develop high blood sugar levels during pregnancy without having diabetes. Evidence on the effects of probiotics on the blood glucose levels of women with GDM is inconsistent. *Objective:* The present study aimed to investigate the effects of probiotics on the blood glucose levels of pregnant women. *Methods:* Online databases, such as PubMed, Cochrane, and Excerpta Medica Database (EMBASE) were searched for randomized controlled trials (RCTs) published before July 2018. Trials had to meet the inclusion criteria of our study. Methodological quality and risk bias were independently assessed by two reviewers. Data were pooled using a random effects model and were expressed as the mean difference (MD) and 95% confidence interval (CI). Heterogeneity was evaluated and quantified as *I*^2^. *Results:* In total, 12 RCTs were included in this study. Studies have shown that the use of probiotics significantly reduced the fasting blood glucose (FBG) level (MD: −0.10 mmol/L; 95% CI: −0.19, −0.02), insulin concentration (MD: −2.24 μIU/mL; 95% CI: −3.69, −0.79), Homeostasis Model Assessment of Insulin Resistance (HOMA-IR) score (MD: −0.47; 95% CI: −0.74, −0.21), and Homeostasis model of assessment-estimated β cell function (HOMA-B) score (MD: −20.23; 95% CI: −31.98, −8.49) of pregnant women. In a subgroup analysis, whether the blood glucose-lowering effect of probiotics influenced the diagnosis of pregnant women with GDM was assessed. The results showed that probiotics had significantly reduced the fasting blood glucose (FBG) level (MD: −0.10 mmol/L; 95% CI: −0.17, −0.04) and HOMA-IR score (MD: −0.37; 95% CI: −0.72, −0.02) of pregnant women who were not diagnosed with GDM. *Conclusion:* Probiotics reduce the blood glucose level of pregnant women, especially without GDM diagnosis. However, further research using RCTs must be conducted to validate the results of the present study.

## 1. Introduction

Gestational diabetes mellitus (GDM) is defined as any degree of glucose intolerance with onset or first recognition during pregnancy [1], but without a known history of diabetes. Several complications may occur during pregnancy due to poor glycemic control. GDM is associated with a range of adverse pregnancy outcomes for the mother, such as pre-eclampsia (high blood pressure with protein in the urine) and instrumental or operative delivery [2]. Because the mother’s blood goes through the placenta to the fetus through the blood vessels in the umbilical cord, the fetus will also be in a state of hyperglycemia and will need to secrete more insulin to absorb the mother’s blood sugar. The fetus will be overweight and will have larger shoulder and extremity circumferences [3,4]. GDM can cause the following conditions in a fetus or newborn: Congenital malformation, fetal death, macrosomia [5,6], neonatal respiratory distress syndrome [7], neonatal hypoglycemia, neonatal jaundice, or hypocalcemia [4,8]. Both mothers with GDM and their infants are at increased risk of diabetes mellitus and metabolic dysfunction later in life [9,10]. Dietary and lifestyle interventions still lack strong experimental evidence to demonstrate their effectiveness. The recruitment rates of studies were small and studies showed that lifestyle interventions did not change fasting blood glucose or type 2 diabetes risk [11]. There are many restrictions on the use of blood glucose lowering drugs by pregnant women. There are two pharmacologic options in pregnant patients who require medical therapy aimed at controlling blood glucose: Insulin and selected oral antihyperglycemic agents, such as metformin and glyburide [12]. Pharmacotherapy has benefits for glucose control, but may result in significant side effects, including abdominal discomfort, dizziness, diarrhea, and hypoglycemia [13]. Therefore, it is important to prevent pregnancy-induced hyperglycemia in pregnant women.

Probiotics are living microorganisms that are beneficial to human health [14,15,16]. Some studies have shown that probiotics can improve immune function [17], relieve irritable bowel syndrome, lower blood pressure [18] and lipid levels [19], and contribute to glycemic control [20,21,22]. Several studies have shown that gut microbes are associated with diabetes and metabolic diseases [23,24]. Studies have found that intestinal microbes change during pregnancy and that probiotics can alter intestinal microbes. Because probiotics improve glycemic control and gut microbes may be associated with diabetes, probiotics can alter gut microbes. The question now is whether probiotics can lower the blood sugar levels of pregnant women. However, the results of numerous human trials were inconsistent [25,26,27,28,29,30,31,32,33,34,35,36,37]. For example, a randomized controlled trial (RCT) conducted by Dolatkhah et al. demonstrated that probiotics can significantly improve blood glucose levels after at least six weeks of use [27]. In addition, Wickens et al. [34] described a broadline significant reduced fasting blood sugar level in pregnant women by performing an RCT with larger sample sizes. However, in other studies with small sample sizes, the use of probiotics did not have significant hypoglycemic effects during pregnancy [28,29,30,31,32,33,35,36,37]. The reason for the non-significant outcome of these studies could be due to the smaller sample sizes. Combing studies in meta-analyses increases the sample size and produces more precise estimates of the effect size than a single RCT. Based on the inconsistency and small sample size of these studies, we conducted a meta-analysis of the data available up to date to investigate the efficacy of probiotics in lowering the blood glucose levels of pregnant women. The present study aimed to assess the effects of probiotics on the blood glucose levels of pregnant women based on the results of previous RCTs.

## 2. Methods

### 2.1. Literature Search

Online databases, such as PubMed, Cochrane, and Excerpta Medica Database (EMBASE), were searched for relevant literature published until July 2018. The keywords used were as follows: Probiotics OR bifidobacteria OR lactobacillus OR streptococcus OR saccharomyces AND diabetes OR glycemic OR glucose OR insulin AND pregnant. T.-R.P. and T.-W.W. conducted independent literature searches. Duplicated studies were excluded, and relevant studies were searched based on screening titles, abstracts, and full texts. The detailed information of the search strategy for eligible studies is presented in the flow diagram provided by Systematic Reviews and Meta-Analyses (PRISMA) [38].

### 2.2. Inclusion Criteria

Search criteria were limited to studies written in English and those that involved humans and pregnant women. Clinical trials had to meet the following criteria: (1) RCTs, (2) focus on adults ≥16 years with or without gestational diabetes, (3) probiotic products used in their intervention group, and (4) one or all of the following data were included: Fasting plasma glucose level, fasting blood glucose (FBG) level, insulin concentration, insulin resistance, Homeostasis Model Assessment of Insulin Resistance (HOMA-IR) score (steady-state model for assessing insulin resistance), and Homeostasis Model Assessment (HOMA-B) score (HOMA for β-cell function).

### 2.3. Data Extraction

Data and decisions were extracted and recorded independently by the two reviewers. The results were then compared, and disagreements were resolved by a third reviewer. The extracted data included the following: Author, year of publication, study design, population, sample sizes, intervention, duration, and outcome.

### 2.4. Quality Assessment

The two reviewers independently assessed the methodological quality of each study using the risk of bias method recommended by the Cochrane Collaboration [39]. Several domains were assessed, including the adequacy of the randomization, allocation concealment, blinding of the patients and outcome assessors, duration of the study (the trial duration of probiotics), information provided to the patients regarding study withdrawals, whether intention-to-treat analysis was performed, and freedom from other biases.

### 2.5. Statistical Analyses

Statistical analysis was performed according to the Cochrane Handbook for Statistical Review of Interventions (version 5.1) [40]. The meta-analysis was performed using RevMan software (Cochrane Review Manager Version 5.1, Oxford, UK) and Comprehensive Meta-Analysis software V2 software. Treatment effects and 95% confidence interval (CI) were calculated using the mean difference (MD). Heterogeneity was assessed with the Chi-square test and *I*^2^ statistics. A *p*-value < 0.10 or *I*^2^ > 50% indicates that heterogeneity existed among the studies. The random-effects model was used. Potential publication bias was assessed using the Funnel plot and Egger’s regression test. A *p*-value > 0.05 based on the Egger’s regression test indicated the absence of publication bias.

## 3. Results

### 3.1. Characteristics of the Included Studies

**Figure 1:**
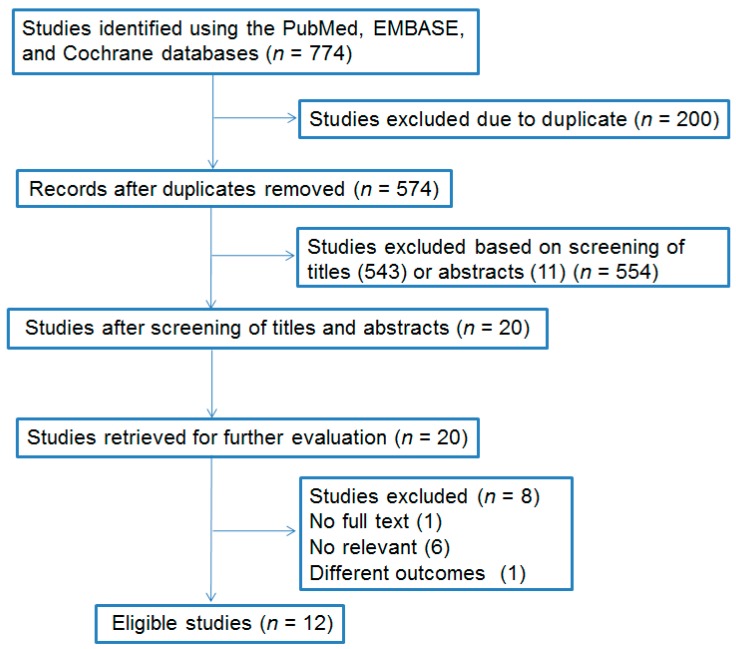
Flowchart describing the inclusion of studies. Excerpta Medica Database (EMBASE).

After the search, 774 studies were included. After the removal of duplicate publications and exclusion of irrelevant articles, the meta-analysis included 12 RCTs involving 1196 pregnant women. The flow chart of the meta-analysis article selection is shown in Figure 1. Table 1 and Table 2 depict information about the studies, including specific information and quality assessment results.

### 3.2. FBG Level

**Figure 2:**
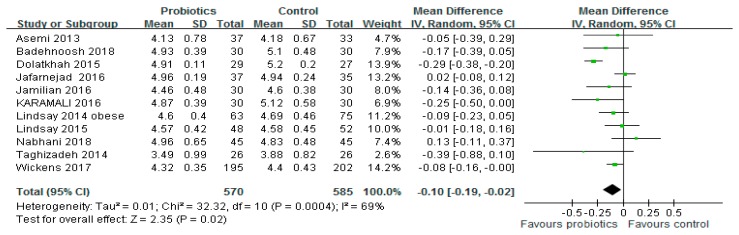
Effect of probiotics on fasting plasma glucose (mmol/L) in pregnant women. The mean difference (MD) and 95% CIs are presented graphically by a square box and horizontal line. The diamond represents the overall MD with its 95% CI using a random effects model.

Figure 2 shows a forest plot of the combined effects of probiotics on fasting blood glucose (FBG) levels. Eleven studies (*n* = 1155) reported changes in FBG levels. This test showed a significant reduction in FBG by 0.10 mmol/L in the intervention group compared with the control group (95% CI: −0.19, −0.02; *p* = 0.02). However, significant evidence of inter-study heterogeneity was observed (*I*^2^ = 69%, *p* < 0.001).

### 3.3. Insulin Resistance

**Figure 3:**
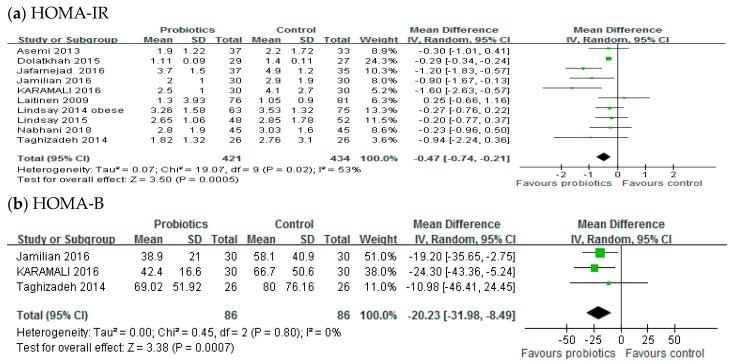
Effect of probiotics on (**a**) HOMA-IR and (**b**) HOMA-B in pregnant women. HOMA-IR: Homeostasis model assessment insulin resistance, HOMA-B: Homeostasis model of assessment-estimated β cell function. The mean difference (MD) and 95% CIs are presented graphically by a square box and horizontal line. The diamond represents the overall MD with its 95% CI using a random effects model.

Ten studies, with a total of 855 participants, reported the effects of probiotics on the HOMA-IR score. A meta-analysis of 10 trials showed a significant reduction in the mean difference of the HOMA-IR score (MD: −0.47; 95% CI: −0.74, −0.21; *p* < 0.001) of the intervention group compared to the control group, as shown in Figure 3a. However, significant inter-study heterogeneity was observed in the overall analysis (*I*^2^ = 53%, *p* = 0.02). Only three trials (*n* = 172) reported the effect of probiotics on the HOMA-B score. The HOMA-B score of the probiotic group was significantly different from that of the control group. The aggregated mean difference was −20.23 (95% CI: −31.98, −8.49; *p* < 0.001), as shown in Figure 3.

### 3.4. Insulin Concentration

**Figure 4:**
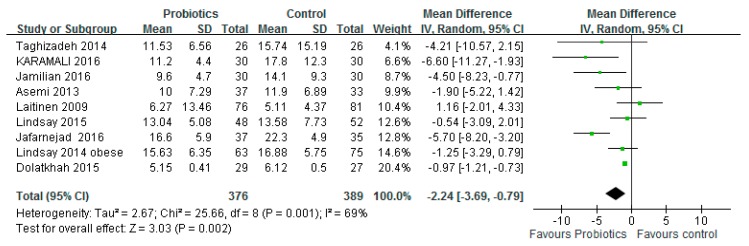
Effect of probiotics on insulin concentration (μIU/mL) in pregnant women. The mean difference (MD) and 95% CIs are presented graphically by a square box and horizontal line. The diamond represents the overall MD with its 95% CI using a random effects model.

Nine studies (*n* = 765) reported changes in insulin concentrations after the intake of probiotics or placebo. Figure 4 shows a forest map of the combined effect of probiotics on insulin concentration. The mean difference in pooling was −2.24 μIU/mL (95% CI: −3.69, −0.79; *p* = 0.002). Significant evidence of heterogeneity among studies was observed (*I*^2^ = 69%, *p* = 0.001).

### 3.5. Subgroup Analysis, Sensitivity, and Publication Bias

**Figure 5:**
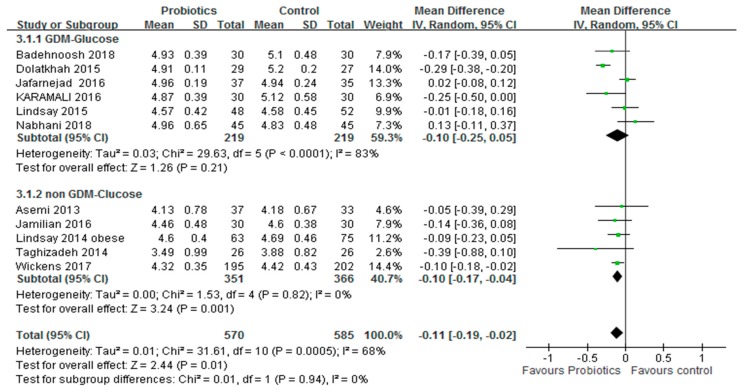
Subgroup analyses for pregnant women diagnosis with GDM or insulin resistance on FBG. GDM: Gestational diabetes mellitus, FBG: Fasting blood glucose. The mean difference (MD) and 95% CIs are presented graphically by a square box and horizontal line. The diamond represents the overall MD with its 95% CI using a random effects model.

**Figure 6:**
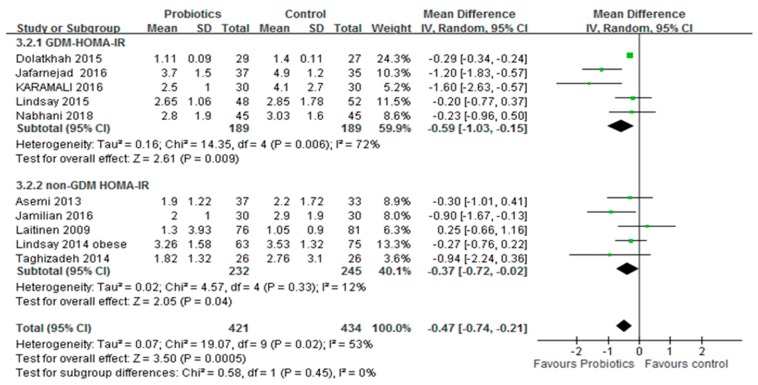
Subgroup analyses for pregnant women diagnosis with GDM or insulin resistance on HOMA-IR. GDM: Gestational diabetes mellitus, HOMA-IR: Homeostasis model assessment insulin resistance. The mean difference (MD) and 95% CIs are presented graphically by a square box and horizontal line. The diamond represents the overall MD with its 95% CI using a random effects model.

Subgroup analysis was performed on pregnant women with or without GDM. Results showed that probiotics had significant effects on the FBG level and HOMA-IR score of pregnant women who were not diagnosed with GDM. The summary results showed no significant reduction in the FBG level of pregnant women after diagnosis of GDM. However, probiotics improved the HOMA-IR score. The summary results are shown in Figure 5 and Figure 6.

Sensitivity analysis of systematically removing individual tests showed that the heterogeneity of the removal test was high and that the Taghizadeh trial [28] may be a heterogeneous study in that meta-analysis. When the study by Taghizadeh was removed from this meta-analysis, there was no evidence of heterogeneity in other FBG studies.

**Figure 7:**
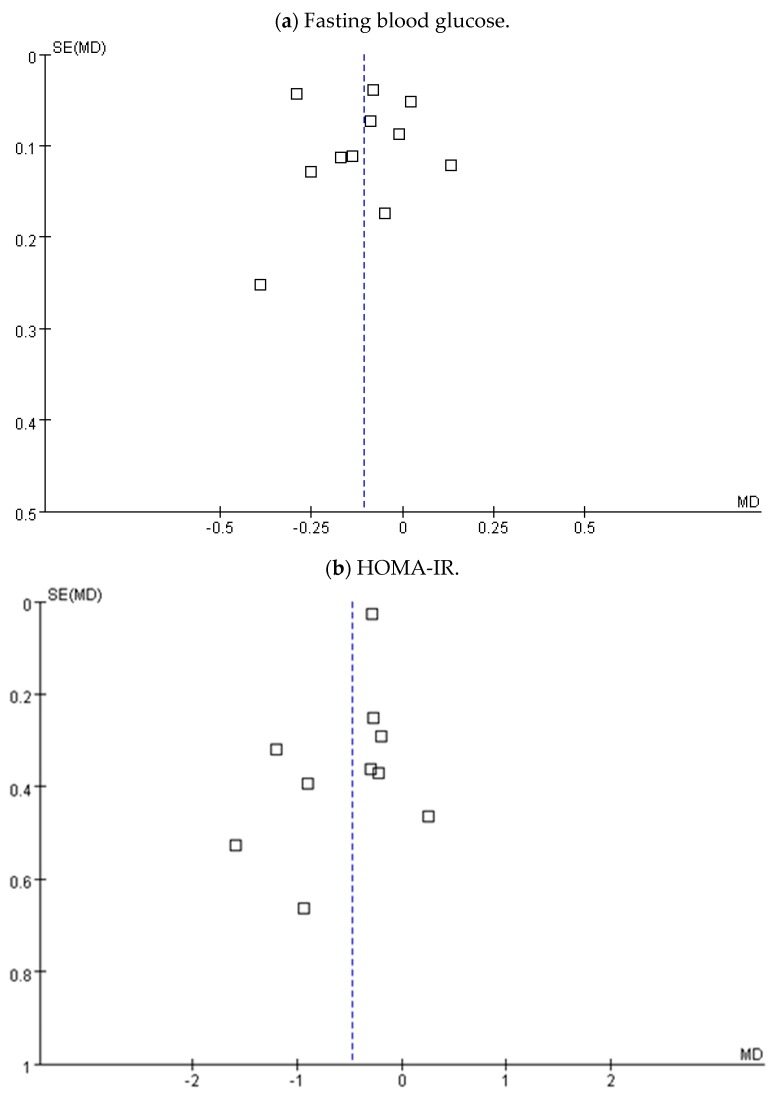

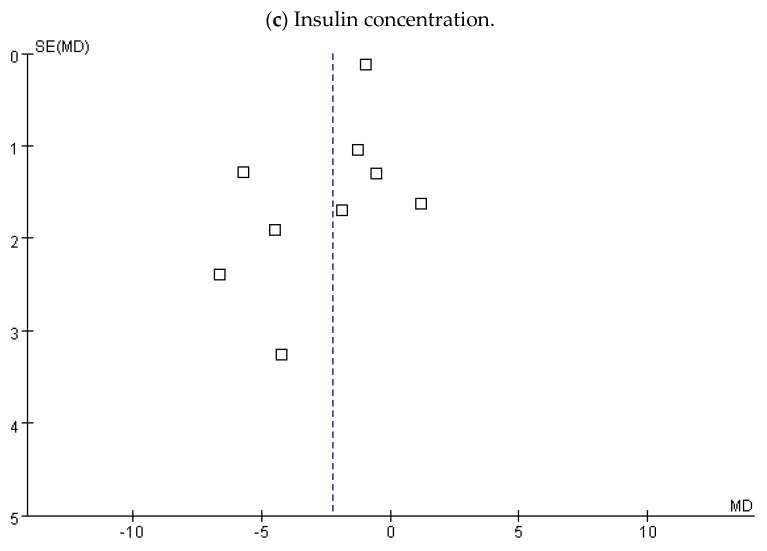
Publication bias funnel plots for (**a**) fasting blood glucose, (**b**) HOMA-IR, and (**c**) insulin concentration. HOMA-IR: Homeostasis model assessment insulin resistance.

The Funnel plot test showed no clear evidence of the FBG level, HOMA-IR score, and insulin concentration bias, as shown in Figure 7. The Egger’s regression test did not show significant publication bias for FBG levels, with a *p* value of 0.408.

## 4. Discussion

According to a previous study, pregnant women with a fasting plasma glucose level of 100 to 105 mg/dL were associated with a five-fold greater risk of macrosomia than those with a fasting glucose level less than 75 mg/dL [4]. Therefore, adequate glycemic control among pregnant women is important. The results of this meta-analysis showed probiotics use in pregnant women could reduce FBG levels (0.10 mmol/L, 95% CI: −0.19, −0.02; *p* = 0.02), the HOMA-IR score (−0.47; 95% CI: −0.74, −0.21; *p* < 0.001), and the HOMA-B score (−20.23; 95% CI: −31.98, −8.49; *p* < 0.001). Those results prove that probiotics have beneficial effects on glycemic control in pregnant women.

Several studies reported that probiotics may regulate glucose metabolism and metabolic syndrome [42,43,44], and the regulation of glucose metabolism is associated with improvement in type 2 diabetes and hyperglycemia. However, hyperglycemia caused by pregnancy is a special case. Women who are not diabetic or obese before pregnancy can develop insulin resistance due to changes in hormones or intestinal flora or weight gain during pregnancy, and the condition may even develop into GDM. Studies have shown that the incidence rate of GDM can be as high as 14% [45]. The composition changes of the intestinal flora during pregnancy may affect the metabolic function of the host. Probiotic supplementation during pregnancy may help maintain the density of the intestinal flora, thereby reducing the metabolic imbalance in pregnant women [46,47].

A systematic review and meta-analysis showed that probiotics can improve the FBG level, insulin concentration, and the HOMA-IR score of patients ≥18 years of age with or without diabetes. Moreover, probiotics have a moderate controlling effect on blood glucose levels. However, these microorganisms are more effective in reducing the FBG level of patients with diabetes and the blood glucose level of those without diabetes [44]. The result of glucose reduction of the probiotics group compared with that of the control group was similar with a previous study [44]. Taylor et al. assessed four RCTs that included 288 pregnant women with GDM. Results demonstrated that probiotics did not improve the FBG or low-density lipoprotein cholesterol (LDL-C) level of pregnant women. However, probiotics may reduce insulin resistance [25]. The discrepancy in the results of these meta-analyses could be due to different studies being included. A well-designed randomized controlled trial is needed to elucidate the benefit effects of probiotics in FBG. In our study, we included studies that focused on pregnancy women with or without gestational diabetes due to the small sample size of these studies. Our results showed that probiotics improved the FBG level, HOMA-IR score, and insulin concentration of women who were diagnosed with GDM and those who were only insulin resistant.

In our subgroup analysis, the controlling effect of probiotics on the FBG level and HOMA-IR score of pregnant women diagnosed with GDM and those who only had insulin resistance were examined separately. In relation to this, we think that probiotics may prevent the development of GDM among pregnant women and that probiotics are beneficial for pregnant women with GDM. The subgroup analysis results showed that probiotics improved the FBG level and HOMA-IR score of pregnant women without GDM diagnosis. These may be associated with women diagnosed with GDM having high level insulin resistance and decreased insulin secretory capacity compared with non-GDM [48]. In a study conducted by Jafarnejad et al., the mean baseline FBG of the probiotic group of pregnancy women with GDM was 89.3 mg/mL [32]. However, in a study conducted by Jamilian et al., the mean baseline FBG of a probiotic group of pregnancy women without GDM diagnosis was 80.3 mg/mL [35]. Therefore, the effect of probiotics for lowering FBG in pregnant women diagnosed with GDM is minimal. There was evidence of substantial inter-study heterogeneity in the overall effect for FBG, insulin, and HOMA-IR. Therefore, more subgroup analyses are needed in the future, such as species, duration, daily dose, and source of probiotics. These may have important ramifications on the effects observed and help explain the heterogeneity across the studies.

The present study has some limitations. First, this study did not obtain data from unpublished trials, which may have led to some publication bias. However, the Funnel plot and Egger’s regression test showed no significant publication bias (*p* = 0.408). Second, the use of strains and duration of probiotics were not consistent across studies that were included in this analysis. To provide more reliable and accurate results to assist medical professionals in making clinical decisions related to the prevention and treatment of GDM, more high-quality RCTs must be conducted. Researchers should further analyze and report findings, such as classification of the species, flora count of the probiotics, duration of probiotic treatment, and dosage.

## 5. Conclusions

The present meta-analysis found that probiotic supplementation resulted in a significant reduction in FBG, insulin resistance, and insulin concentration in pregnant women, especially without GDM diagnosis. However, more rigorous RCTs must be conducted to validate the results of the present study.

## Figures and Tables

**Table 1:** Characteristics of studies included in the meta-analysis.

Study	Design	Intervention/Control (Sample Size)	Age	Duration (Weeks)	Probiotic	Probiotic Source	Dose (CFU)	Outcomes
Asemi et al. [29]	SB	Probiotic yogurt/Conventional yogurt (37/33)	18–30	9	*L. acidophilus*, *L. bulgaricus*, *S. thermophiles*, *B. animals*	Y	1 × 10^7^	FBG HOMA-IR Insulin
Laitinen et al. [41]	DB	Probiotic/placebo (66/70)	25–35	20	*L. rhamnosus*, *B. lactis*	C	1 × 10^10^	HOMA-IR Insulin
Lindsay et al. [30]	DB	Probiotic/placebo (63/75)	26–36 OB	4	*L. salivarius*	C	1 × 10^9^	FBG HOMA-IR Insulin
Karamali et al. [31]	DB	Probiotic/placebo (30/30)	18–40	6	*L. acidophilus*, *L. casei*, *B. bifidum*	C	6 × 10^9^	FBG HOMA-IR Insulin HOMA-B
Dolatkhah et al. [27]	DB	Probiotic/placebo (29/27)	18–45	8	*L. acidophilus*, *Bifidobacterium*, *S. thermophiles*, *L. bulgaricus*	C	4 × 10^9^	FBG HOMA-IR Insulin
Jafarnejad et al. [32]	DB	Probiotic/placebo (37/35)	32.4 ± 3.1, 31.9 ± 4.0	8	*B. longum*, *B. infantis*, *L. acidophilus*, *L. plantarum*, *L. paracasei*, *L. delbrueckii* subsp. Bulgaricus	C	15 × 10^9^	FBG HOMA-IR Insulin
Lindsay et al. [33]	DB	Probiotic/placebo (48/52)	>18	6	*L. salivarius*	C	1 × 10^9^	FBG HOMA-IR Insulin
Taghizadeh et al. [28]	TB	Synbiotic/placebo (26/26)	18–35	9	*L. sporogenes*	C	18 × 10^7^	FBG HOMA-IR Insulin HOMA-B
Wickens et al. [34]	DB	Probiotic/placebo (195/202)	>16	>12	*L. rbamnosus* HN001	C	6 × 10^9^	FBG
Jamilian et al. [35]	DB	Probiotic/placebo (30/30)	18–37	12	*L. acidophilus*, *L. casei*, *B. bifidum*	C	6 × 10^9^	FBG HOMA-IR Insulin HOMA-B
Badehnoosh et al. [36]	DB	Probiotic/placebo (30/30)	18–40	6	*L. acidophilus*, *L. casei*, *B. bifidum*	C	6 × 10^9^	FBG
Nabhani et al. [37]	DB	Symbiotic/placebo (45/45)	18–40	6	*L. acidophilus*, *L. plantarum*, *L. fermentum*, *L. gasseri*	C	2.5 × 10^10^ 7.5 × 10^9^ 3.5 × 10^9^ 1 × 10^10^	FBG HOMA-IR

C: Capsule, Y: Yogurt, D: Drink, OB: Obesity, HN001: *Lactobacillus rhamnosus* HN001, CFU: Colony-forming unit, SB: Single blind, DB: Double blind, TB: Triple blind, FBG: fasting blood glucose, HOMA-IR: Homeostasis model assessment insulin resistance, HOMA-B: Homeostasis model of assessment-estimated β cell function.

**Table 2:** The quality assessment of the 10 randomized controlled trials included.

Reference	Adequate Sequence Generation	Allocation Concealment	Blinding	Incomplete Outcome Data Addressed	Free of Selective Reporting	Free of Other Bias *
Asemi et al. [29]	Yes	Unclear	Yes	Yes	Yes	Yes
Laitinen et al. [41]	Yes	Yes	Yes	Yes	Yes	Yes
Lindsay et al. [30]	Yes	Yes	Yes	Yes	Yes	Unclear
Karamali et al. [31]	Yes	Yes	Yes	Yes	Yes	Unclear
Dolatkhah et al. [27]	Yes	Yes	Yes	Yes	Yes	Unclear
Jafarnejad et al. [32]	Yes	Yes	Yes	Yes	Unclear	Unclear
Lindsay et al. [33]	Yes	Yes	Yes	Yes	Yes	Unclear
Taghizadeh et al. [28]	Yes	Yes	Yes	Yes	Yes	Unclear
Wickens et al. [34]	Yes	Yes	Yes	Yes	Yes	Unclear
Jamilian et al. [35]	Yes	Yes	Yes	Unclear	Yes	Unclear
Badehnoosh et al. [36]	Yes	Yes	Yes	Yes	Unclear	Unclear
Nabhani et al. [37]	Yes	Yes	Yes	Yes	Yes	Unclear

Note: * Other bias refers to selective bias and measurement bias.
